# Evaluation of alveolar cortical bone thickness and density for orthodontic mini-implant placement

**DOI:** 10.4317/jced.51228

**Published:** 2013-12-01

**Authors:** Michele Cassetta, Aisha AA. Sofan, Federica Altieri, Ersilia Barbato

**Affiliations:** 1 DDS, PhD. Assistant Professor, Department of Oral and Maxillofacial Sciences, School of Dentistry, “Sapienza” University of Rome, Italy; 2 DDS, PhD. Orthodontist, Department of Oral and Maxillofacial Sciences, Al- Thawra Modern General Hospital, Sanaa, Yemen; 3 DDS. Assistant Researcher, Department of Oral and Maxillofacial Sciences, School of Dentistry, “Sapienza” University of Rome, Italy; 4 DDS, MS. Professor, Department of Oral and Maxillofacial Sciences, School of Dentistry, “Sapienza” University of Rome, Italy

## Abstract

Objective: Mini-implant stability is primarily related to bone quality and quantity. This study evaluated alveolar cortical bone thickness and density differences between interradicular sites at different levels from the alveolar crest, and assessed the differences between adolescents (12-18 years of age) and adults (19-50 years of age), males and females, upper and lower arch, anterior and posterior region of jaws and buccal and oral side. Study Design: In this retrospective study, 48 Computed Tomography scans, performed for oral surgery purposes were selected from dental records of 3,223 Caucasian orthodontic patients. The SimPlant software (Materialise, Leuven, Belgium) was used to measure cortical bone thickness and density at 13 interradicular sites and four bone levels ( 2,4,6 and 8 mm ). For the statistical analysis descriptive statistics, Student’s t-test and Pearson correlation coefficient were used. Results: Statistically significant differences in alveolar cortical bone thickness and density between age, gender, sites and sides were found (P<0.05). The Pearson correlation coefficient demonstrated a significant linear increasing of thickness and density from crest to base of alveolar crest (P≤0.05). Conclusion. Adults show a thicker alveolar cortical bone than adolescents. Alveolar cortical bone thickness and density were greater in males than in females, in mandible than in maxilla, in the posterior region than the anterior, in oral than buccal side. There is an increase of thickness and density from crest to base of alveolar crest.

** Key words:**Orthodontics, cortical bone thickness, cortical bone density, mini-implant, computed tomography, temporary anchorage devices.

## Introduction

Strategies for anchorage control have been a major factor in achieving successful orthodontic treatment. In recent years, mini-screws implants, also called Temporary Anchorage Devices (TAD), are increasingly being used to provide intraoral orthodontic anchorage. Several studies found that the stability of TAD is affected by age, sex, craniofacial skeletal pattern, site and side of implantation, latent period, loading protocol, dimension and angulation of TAD, insertion torque, degree of TAD-bone contact, quality and quantity of the cortical bone, degree of inflammation of the peri-TAD-tissue, thickness and mobility of the soft tissue, and root proximity (1-6). For these reasons, research has been conducted on the stability of mini-implants used for orthodontic purposes.

Recent studies have evaluated the cortical bone thickness for mini-implant placement in patients (7-10) and skulls (11-14) using computerized tomography (CT). Concerning the alveolar cortical bone density few studies exist in literature (15-18).

Knowledge of the buccal and oral cortical bone thickness and density in various areas should guide clinicians in selecting the placement site and the proper placement and loading protocols. The purpose of this study was to examine the alveolar cortical bone thickness and density for TAD placement and to evaluate the differences between age, sex, site and side of implantation using CT. The hypotheses were that there are no differences in alveolar cortical bone thickness and density between, males and females, adolescents and adults, upper and lower arch, anterior and posterior area of the jaws, between buccal and oral side and from crest to base of alveolar crest.

## Material and Methods

In this retrospective study, 48 Computed Tomography scans, performed for oral surgery purposes, were selected from dental records of 3,223 Caucasian orthodontic patients.

The study sample was divided into groups based on age (adolescent:12-18 years; adults:19-50 years), gender (males and females), site (upper and lower arch; anterior or incisive-canine region and posterior or premolar-molar region) and side (buccal and oral or palatal/lingual).

The scans were selected according to the following inclusion criteria:

● All erupted permanent teeth in the quadrant measured.

● Absence of periapical or periradicular pathologies or radiolucencies of either periodontal or endodontic origin.

● No significant medical or dental history (e.g. use of bisphosphonates or bone-altering medications).

● No severe facial or dental asymmetries.

● Absence of vertical or horizontal periodontal bone loss.

**Figure 1:**
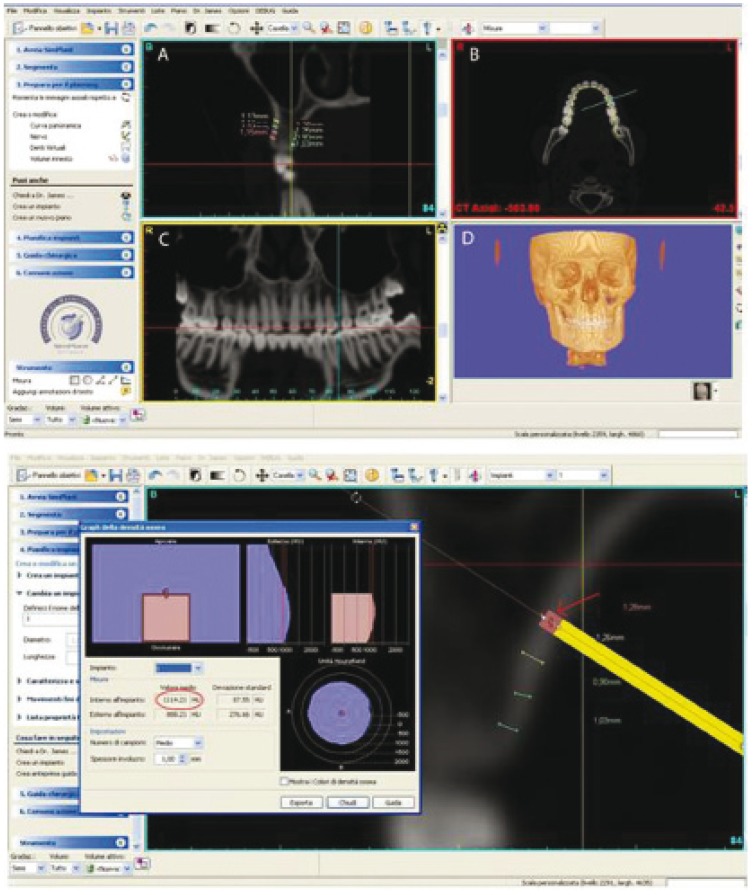
The computer program employed in the present study (SimPlant® - Materialise-Leuven-Belgium) allows visualization of inter-radicular spaces in a multitude of 2-D and 3-D points of view. The cross-sectional (A), axial,(B), panoramic (C) and 3-D (D) images are visible at the same time on a computer monitor. Lower image: The cross-section image from SimPlant used to measure the cortical bone thickness at 2, 4, 6 and 8 mm from the alveolar crest.

The data were obtained with a spiral multisliced Asteion Multi CT system (Toshiba Medical Systems). The CT parameters used were 0° gantry tilt, high resolution bone Kernel, 0,5 mm nominal slice thickness, 0,5 mm interval and 0,5 mm pitch. In this study, data processing and all measurements were performed by SimPlant software (Materialise, Leuven, Belgium), a program that allows a 3D reconstruction and images of anatomical structures along planes and curves from data acquired with CT. With this program CT images are imported; then a 3D reconstruction was created from the 2D CT images, and the panoramic curve was obtained. The following four view windows were selected: 2D cross sectional slices; 2D axial slices; 2D panoramic slices and 3D image (Fig. 1).

The cortical bone thickness and density were measured at 2, 4, 6 and 8 mm intervals apical to the alveolar crest at thirteen interradicular sites form the right 2nd molar to the left 2nd molar in both maxilla and mandible.

Before measuring the alveolar cortical bone thickness, each site was oriented in all view windows. The panoramic and axial views were used to locate the interradicular area of interest. The cross-section image (perpendicular to the panoramic curve), simultaneously visible in the computer monitor interactive window, was used to perform four measurements at 2, 4, 6 and 8 mm from the alveolar crest (Fig. 1).

The software was used also to measure bone density at the same levels (2, 4, 6 and 8 mm from the alveolar crest). An area of 1 mm2 was used to calculate the density of alveolar cortical bone. Bone density was measured using Hounsfield units (HU), which are directly associated with tissue attenuation coefficients. Density value was measured when cortical bone thickness was ≥ 1 mm.

The reproducibility of method was assessed by re-examining the records of 10 patients 2 weeks after the first examination by a single operator. Reproducibility was 98% for thickness measurements and 97% for density measurements.

Statistical analysis was conducted at site level. For the statistical analysis, data were evaluated using SPSS software (Statistical Package for Social Science, IBM Corporation, NY-USA). Quantitative data of each group was described, with mean and minimum-maximum values. Alveolar cortical bone thickness values were illustrated using box plots showing median, quartile, and extreme values. Considering the thickness and density values, the T-test was used to determine the influence of age, gender, site and side. The significance was set at P≤0.05. The Pearson correlation coefficient was used to evaluate thickness and density increasing from crest to base of alveolar crest. Again, the threshold for significance was set at P≤0.05.

The local ethical committee was informed about the study protocol. We have read the Helsinki Declaration and followed the guidelines in the present investigation.

## Results

The average and minimum-maximum values of the cortical bone buccal thickness and of the cortical bone oral thickness, at different levels from the alveolar crest, are shown in  Table 1 and  Table 2. In  Table 3 and  Table 4 are shown the average and minimum-maximum values of cortical bone buccal density and of cortical bone oral density. All Tables ( Table 1, Table 2, Table 3, Table 4) showed a gradual increase of mean values and the Pearson correlation coefficient demonstrated that there was a significant linear increasing of thickness and density from crest to base of alveolar crest (P≤0.05).

**Table 1:**
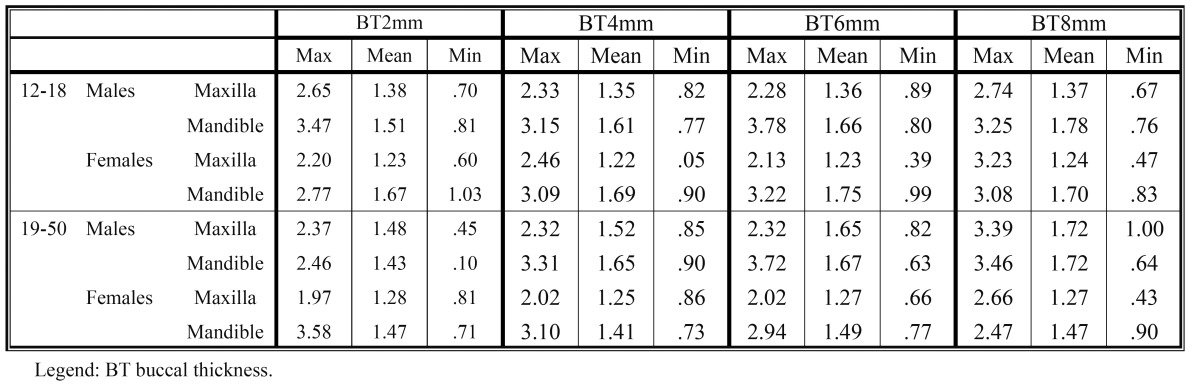
The mean, minimum-maximum of buccal cortical bone thickness values (mm) at different levels (2, 4, 6, 8 mm) from the alveolar crest.

**Table 2:**
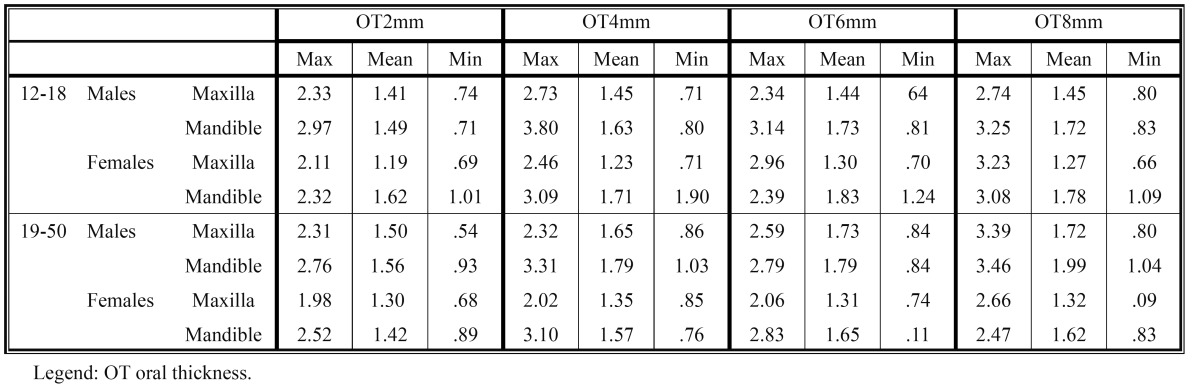
The mean, minimum-maximum of oral cortical bone thickness values (mm) at different levels (2, 4, 6, 8 mm) from the alveolar crest.

**Table 3:**
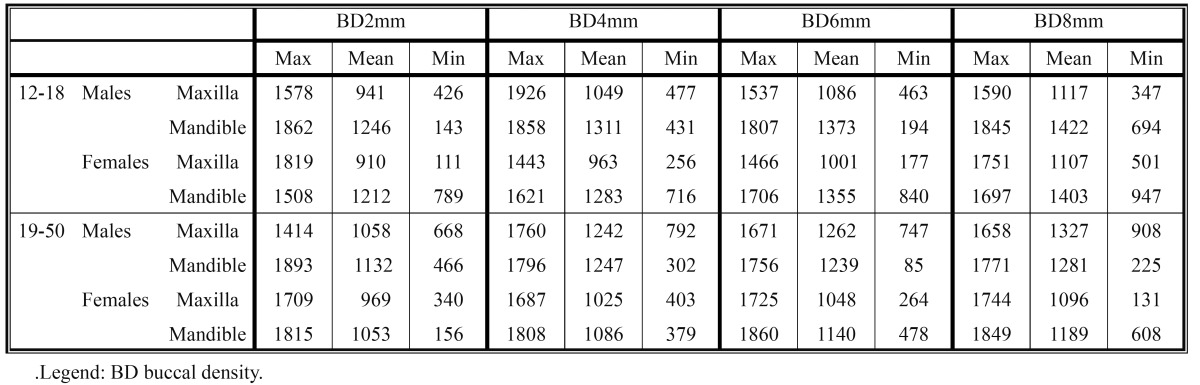
The mean, minimum-maximum of buccal cortical bone density values (HU) at different levels (2, 4, 6, 8 mm) from the alveolar crest.

**Table 4:**
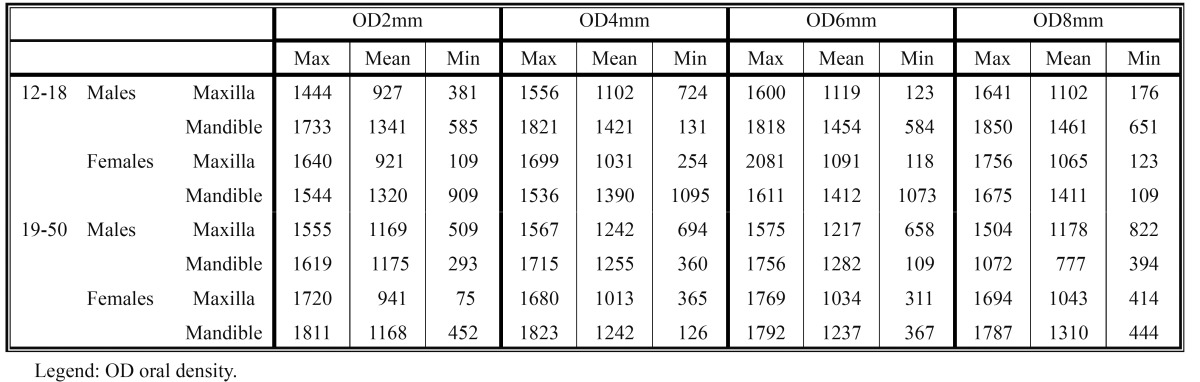
The mean, minimum-maximum of oral cortical bone density values (HU) at different levels (2, 4, 6, 8 mm) from the alveolar crest.

**Figure 2:**
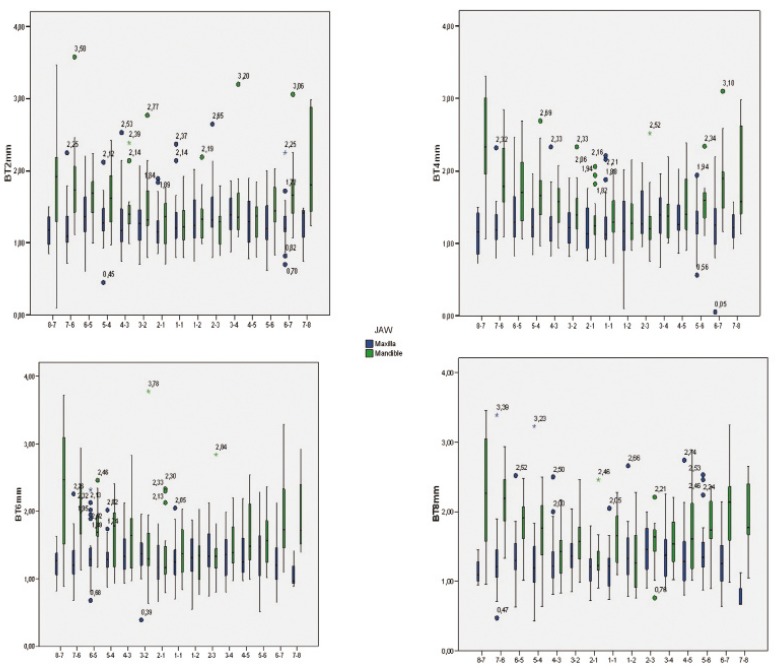
Box-plots show median, quartile, and extreme values of bone thickness ( in millimeters ). Boxes include 50% of values; the horizontal lines inside the box indicate the medians, and the vertical lines extend to 1.5 of the inter-quartile range. Circles depict outliers.

**Figure 3:**
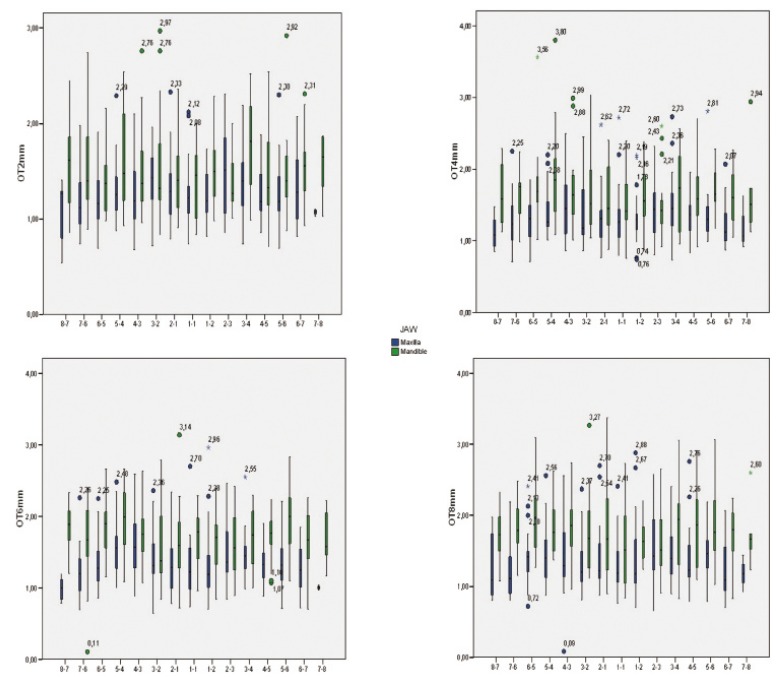
Box-plots show median, quartile, and extreme values of bone thickness ( in millimeters ). Boxes include 50% of values; the horizontal lines inside the box indicate the medians, and the vertical lines extend to 1.5 of the inter-quartile range. Circles depict outliers.

The thickness data, evaluating each site and level of measurement, were also illustrated using box plots (Fig. 2,3).

In males higher values of thickness and density than females were found, at different levels (2 mm, 4 mm, 6 mm, 8 mm) from the alveolar crest, with a statistically significant difference (P≤0.05).

In adults, the thickness of both jaws was greater than in adolescents with a statistically significant difference at 4, 6, 8 mm from the alveolar crest (P≤0.05). The density values did not show any difference according to the age of patients; only at 8 mm cortical oral bone level, the adolescents recorded higher density values with a statistically significant difference (P≤0.05).

The lower jaw was found both thicker and higher in density than the upper jaw with a statistically significant difference (P≤0.05).

Regarding the anterior and the posterior regions of the jaws, thickness values were higher in posterior region than in anterior with a statistically significant difference at 2, 4, 6 and 8 mm form the alveolar crest in the buccal cortical bone side (P≤0.05). Concerning density values the results were ambiguous with statistically significant higher density values, in the posterior region, only in the buccal cortical bone side at 4 and 6 mm from the alveolar crest (P≤0.05).

The paired comparison between buccal and oral side showed a thicker cortical bone in the oral side of both jaws with a statistically significant difference at 4 and 6 mm from the alveolar crest (P≤0.05).

The density of mandibular lingual cortical bone was found significantly higher at 2, 4, 6 and 8 mm whereas in maxillary palatal cortical bone the density was found higher at 4, 6 and 8 mm (P≤0.05).

The results of this study showed that there are differences in alveolar cortical bone thickness and density between, males and females, adolescents and adults, upper and lower arch, anterior and posterior area of the jaws, between buccal and oral side and from crest to base of alveolar crest.

## Discussion

Several studies have proposed a variety of methods for assessing bone density, but in recent years, the use of a CT scan has been common for preoperative quantitative and qualitative assessment of implant sites, and the Hounsfield Unit (HU) is routinely used to determine the bone density objectively (19,20).

Even more recently, due to the need for less expensive image acquisition protocols or for scanners with lower radiation dose, cone beam CT (CBCT) has been widely employed for oral and maxillofacial imaging, as it seems to provide good spatial resolution, gray density range, and contrast, as well as a good pixel/noise ratio (20).

With CBCT, the dimensional accuracy is also comparable with CT, but unlike CT, the gray density values of the CBCT images (voxel value [VV]) are not absolute. In fact, CT could be calibrated using as a reference the density values of the air (-1,000 HU) and pure water (0 HU); otherwise, CBCT cannot be calibrated, and the values, which are based on the difference of gray scale, are already preset by the manufacturer (21) .

In a recent study (20) the possibility of correlating the gray density values recorded by CT and CBCT was demonstrated; in fact, a correlation between VV of CBCT and HU values of multislice CT was observed. More specifically the conversion ratio between the two gray values was determined and defined equal to 0.7; thus, to convert the CBCT gray values into CT, it is necessary to multiply CBCT values by 0.7.

This conversion ratio, moreover, is approximate and may vary based on the CBCT used.

On the basis of these results, in the present study only CT scans were evaluated.

In this study a statistically significant difference between males and females in alveolar cortical bone thickness and density in both jaws was found. The cortical bone thickness and density were greater in males than in females. These results disagree with other authors who found no sex differences (7,10,15-17). Ono et al. (7) asserted that there is not a significant sex difference regarding the alveolar cortical bone thickness at 4 mm from the alveolar crest but they found that cortical bone was thicker in males than females at vertical levels 1 to 2 mm and 5 to 9 mm apical to alveolar crest in the maxilla. Concerning the alveolar cortical bone density Chun and Lim (16) found no relationships with sex but this may be related to subject age (range 25-35 years). It has been reported that bone densities in Korean females peak around 35 years of age, slowly decrease until 50 years old and then rapidly decrease after 50 years of age. Up to 35 years of age, there are no differences in bone densities between Korean male and female.

As stated by other authors (22,23) the sex difference in cortical bone thickness and density, recorded in the current study, might be expected because males have larger bite forces and masticatory muscles than females.

In the current study, the alveolar cortical bone thickness of both jaws was greater in adults with a statistically significant difference at 4, 6, 8 mm from the alveolar crest. Farnsworth et al. (10) in their study found no age-related change distal to mandibular first molar but they found a statistically significant age-related difference in the maxillary buccal region similar results were found by Fayed et al. (9). Ono et al. (7) showed that cortical thickness mesial to the mandibular first molar and 3 to 8 mm apical to the alveolar crest was significantly thicker in adults than adolescents. Considering the results of the present study, the age does not seem to affect the density values, but comparable data are not available in the literature.

Although well-controlled studies have not been performed, it appears that TAD placed in younger or adolescent patients tend to fail more often than those placed in adults (4,8). Difference in cortical thickness between younger and older patients might be explained by allometry such as proportionate increases in overall body size and the size of the body parts (10).

In the present study it was found that the alveolar cortical bone thickness and density are greater in the mandible than in the maxilla. Same results were found by other authors that report a thicker and higher alveolar cortical bone density in the lower jaw than in the upper (2,7,10,12,15-17). Baumgaertel and Hans (12) observe a thicker buccal cortical bone in mandible.

Choi et al. (15) comparing bone density between the maxilla and mandible showed that the mandible had higher values and these differences were more significant in the posterior area of jaws.

Another interesting finding it was that alveolar cortical bone is thinnest in the anterior regions of both jaws and increases progressively toward the posterior. These results agree with those found by Baumgartel and Hans (12) who found a buccal cortical bone thinnest in the anterior sextants of both jaws and a progressive increase toward the posterior region, except distal to the maxillary second molars, where the buccal cortex average was thin. Farnsworth et al. (10) showed a cortical bone thickness decrease from posterior to anterior region. In our study, bone density showed the same increase from the anterior to the posterior except for the oral side, which had higher values in density in anterior area. Higher bone density values from the anterior to the posterior areas were found in other studies (16,17). Our study suggests that the posterior area may contain denser and thicker cortical bone. This pattern might be explained by the higher functional demands placed on the posterior teeth (24,25).

Concerning the buccal and oral side we found a thicker alveolar cortical bone and higher density in the oral side of both jaws. According to Farnsworth et al. (10) the mandibular buccal cortical bone is significantly thicker than the cortical bone in maxillary buccal, maxillary palatal and lingual regions. Sawada et al. (14) evaluating cortical bone thickness of the upper jaw found that the buccal cortical bone was thinner than the palatal cortical bone. Choi et al. (15) comparing bone density between the buccal and lingual sides in the mandible showed that the lingual side had higher values in the anterior area and vice versa in the posterior area. On the other hand, they did not find differences between the buccal and palatal sides in the maxilla.

In the current study gradual increase in the alveolar cortical bone thickness at different distances from the alveolar crest was found. These results agree with those found by Deguchi et al. (2) and Ono et al. (7) who observed that the cortical bone thickness tends to be thicker at greater heights and thinner at shallow levels. Also Sawada et al. (14) reported a tendency for the superior part of the alveolar process to be thicker than the inferior part. Park et al. (17) found, for the mandible, that all density values of the cortical basal bone were statistically higher than those of the alveolar bone. Our results indicate that bone thickness and density vary with the distance from the alveolar crest in the interradicular sites. According to Chun and Lim (16) mini-implants for orthodontic anchorage may be successfully placed in areas with equivalent bone density up to 6 mm apical to the alveolar crest.

Implant placement in the anterior regions of both jaws should be avoided for several reasons: in this area there is little cortical bone for anchorage of implants and little attached gingiva and there is often lack of sufficient interradicular distances (5).

Several factors affect the success rates of mini-implants: anatomic factors, oral hygiene technique used, design of the mini-implant and force used (1). Among these factors, the anatomy of site, especially the thickness and density of the cortical bone, seems to have a direct effect on success rate (4,15,17).

Alveolar cortical bone thickness and density appear to play an important role when planning a mini-implant placement. It is not indicated to perform a CT scan for mini-implants insertion. The present study evaluated the influence of different variables on alveolar cortical bone thickness and density providing the clinicians with useful data to reach a better primary mini-implant stability.

In conclusion.

● Males are characterized by a thicker and higher density alveolar cortical bone than females.

● Adults show a thicker alveolar cortical bone than adolescents;

● In the mandible the alveolar cortical bone is more compact and thicker than in the maxilla;

● High values of thickness and density characterize posterior regions of both jaws;

● The alveolar oral cortical bone is thicker than the buccal.

● There is a significant linear increasing of thickness and density from crest to base of alveolar crest.
